# Atrial Fibrillation Management Strategies in Routine Clinical Practice: Insights from the International RealiseAF Survey

**DOI:** 10.1371/journal.pone.0147536

**Published:** 2016-01-22

**Authors:** Chern-En Chiang, Lisa Naditch-Brûlé, Sandrine Brette, José Silva-Cardoso, Habib Gamra, Jan Murin, Oleg J. Zharinov, Philippe Gabriel Steg

**Affiliations:** 1 General Clinical Research Center and Division of Cardiology, Taipei Veterans General Hospital, National Yang-Ming University, Taipei, Taiwan; 2 Sanofi, Paris, France; 3 Lincoln, Boulogne-Billancourt, France; 4 Department of Cardiology, Porto Medical School, Hospital São João, Porto, Portugal; 5 University of Monastir and Cardiology A Department, FattoumaBourguiba University Hospital, Monastir, Tunisia–LR12SP16 Cardiothrombosis; 6 Department of Internal Medicine and Cardiology, Comenius University, Bratislava, Slovakia; 7 Functional Diagnostics Department, National Medical Academy of Postgraduate Education, Kyiv, Ukraine; 8 INSERM U-1148, Paris, France; Université Paris-Diderot, Paris, France; Assistance Publique–Hôpitaux de Paris, Hôpital Bichat, DHU FIRE, Paris, France; and NHLI, ICMS, Imperial College, Royal Brompton Hospital, London, United Kingdom; University at Buffalo, UNITED STATES

## Abstract

**Background:**

Atrial fibrillation (AF) can be managed with rhythm- or rate-control strategies. There are few data from routine clinical practice on the frequency with which each strategy is used and their correlates in terms of patients’ clinical characteristics, AF control, and symptom burden.

**Methods:**

RealiseAF was an international, cross-sectional, observational survey of 11,198 patients with AF. The aim of this analysis was to describe patient profiles and symptoms according to the AF management strategy used. A multivariate logistic regression identified factors associated with AF management strategy at the end of the visit.

**Results:**

Among 10,497 eligible patients, 53.7% used a rate-control strategy, compared with 34.5% who used a rhythm-control strategy. In 11.8% of patients, no clear strategy was stated. The proportion of patients with AF-related symptoms (EHRA Class > = II) was 78.1% (n = 4396/5630) for those using a rate-control strategy vs. 67.8% for those using a rhythm-control strategy (p<0.001). Multivariate logistic regression analysis revealed that age <75 years or the paroxysmal or persistent form of AF favored the choice of a rhythm-control strategy. A change in strategy was infrequent, even in patients with European Heart Rhythm Association (EHRA) Class > = II.

**Conclusions:**

In the RealiseAF routine clinical practice survey, rate control was more commonly used than rhythm control, and a change in strategy was uncommon, even in symptomatic patients. In almost 12% of patients, no clear strategy was stated. Physician awareness regarding optimal management strategies for AF may be improved.

## Introduction

Atrial fibrillation (AF) is associated with increased mortality and morbidity, including stroke, heart failure, and impaired quality of life [1]. Despite these potential consequences, whether it is better to restore and maintain sinus rhythm (rhythm-control strategy) or allow AF to continue while controlling ventricular rate (rate-control strategy) remains uncertain, since clinical trials have not demonstrated clear superiority of either strategy [2,3].

While randomized clinical trials represent the highest level of evidence, patient populations recruited for clinical trials are highly selective and might not be truly representative of routine clinical practice. In particular, they are often derived from largely Western European and North American settings and may not reflect the variety of clinical manifestations and management strategies.

Surveys and registries provide complementary data on AF management strategies in clinical practice. Most of the previous information either originates from a single country [4,5], Europe [6], or North America [7], or excludes patients with permanent AF [8]. RealiseAF was a recent, large-scale, international, cross-sectional observational survey of patients with all types (almost half with the permanent form) of AF, encompassing Europe, Asia, North Africa, the Middle East, and Latin America [1]. As such, RealiseAF provides a unique opportunity to examine the management strategy of different types of AF in routine clinical practice in a variety of regions and practice settings.

## Methods

### Design

The design and methods of this survey have been previously published [1]. RealiseAF was an international, cross-sectional, observational survey of 11,198 patients with AF registered at 831 sites in 26 countries from October 2009 to May 2010. Participating countries were Algeria, Azerbaijan, Belgium, Bulgaria, Czech Republic, Egypt, Germany, Hungary, India, Ireland, Italy, Lebanon, Lithuania, Mexico, Morocco, Portugal, Russia, Slovakia, Spain, Sweden, Switzerland, Taiwan, Tunisia, Turkey, Ukraine, and Venezuela.

### Objectives

The primary objectives of this sub-analysis were to (i) describe patients’ characteristics according to AF management strategy prior to the visit; (ii) assess the control of AF and AF-related symptoms according to AF management strategy prior to the visit; (iii) determine the predictors for the selection of AF management strategy at the end of the visit; and (iv) analyze the modification of AF management strategy (overall, and according to control of AF and European Heart Rhythm Association [EHRA] class on the day of the visit).

### Patients

Patients with a history of AF (treated or not, and independent of the rhythm at the time of inclusion), with > = 1 AF episode (documented by standard electrocardiogram [ECG] or by Holter ECG in the previous 12 months) or documented current AF, who provided written informed consent, were enrolled. Exclusion criteria were limited to mental disability (such as dementia or significant cognitive disorders), post-operative AF within 3 months of cardiac surgery, and participation in clinical trials investigating AF or antithrombotics in the previous month.

### Selection of investigators

Participating physicians were randomly selected from a global list of cardiologists and internists (office- and hospital-based) in each country. To remove any bias, the ratio of recruited cardiologists to internists was predetermined to reflect the practice in each country; the list and ratio were validated by national coordinators. In order to maximize recruitment of consecutive patients, the maximum duration of enrollment per center was short (6 weeks). Each investigator was asked to recruit a minimum of 10 patients and a maximum of 30.

### Patient assessment, including AF strategy and control

Data were collected on patient demographics, cardiovascular risk factors (arterial hypertension, dyslipidemia, diabetes mellitus, obesity, family history of premature cardiovascular disease/sudden death, smoking status, and amount of physical activity) and comorbidities (heart failure, coronary artery disease, cerebrovascular disease, peripheral arterial disease, and valvular heart disease), left ventricular ejection fraction measurement within the last 12 months, type of AF, AF management strategy prior to and at the end of the visit. Investigators could choose either rate or rhythm strategy based on their judgement. If no specific strategy was undertaken, "none" (no determined strategy) should be chosen. AF control was defined electrocardiographically as being in sinus rhythm or being in AF with a resting ventricular rate ≤80 beats per minute [bpm] at the time of visit on resting ECG). The New York Heart Association (NYHA) classification of heart failure and EHRA (for arrhythmia-related symptoms) classification [9] were used by the investigators to categorize symptoms.

### Statistical analysis

The details of the determination of sample size have been described previously [1]. Population characteristics were summarized as mean and standard deviation for continuous variables and as count and percentages for qualitative variables. Descriptive data were described according to the AF management strategy used prior to and at the end of the visit. Comparisons between subgroups (rhythm-control vs. rate-control strategies) were made using either the χ^2^ test, Fisher’s exact test for nominal variables, or analysis of variance for quantitative variables. The change in AF management strategy at the end of the visit was also described according to AF control and EHRA classification on day of visit.

To identify factors associated with the choice of AF management strategy at the end of the visit (rhythm-control rather than rate-control strategy), a multivariate stepwise logistic regression (with a significance level of 20% for entering and 5% for retaining the variables in the model) was performed. Variables included age (by class); sex; obesity (body mass index [BMI] > = 30 kg/m^2^); physical activity; smoking status; EHRA AF cardiac symptoms classification; time since first AF diagnosis (by class); type of AF; and history of heart failure (by NYHA class), valvular heart disease, hypertension, cerebrovascular disease, coronary artery disease, diabetes, dyslipidemia, peripheral arterial disease, and hyperthyroidism. Discrimination between models was assessed using c-statistics and calibrated using Hosmer–Lemeshow χ^2^ statistics. The odds ratios and associated 95% confidence intervals (CIs) for choosing a rhythm-control strategy rather than a rate-control strategy were determined; the multivariate analysis was adjusted for country. Analyses were performed using SAS^®^ statistical software, version 9.2 (SAS Institute, Cary, NC, USA).

## Results

### Patient characteristics according to AF management strategy prior to the visit

From October 2009 to May 2010, 831 sites were active in screening 11,198 patients [1]. Overall, 10,497 patients were eligible for analysis (Table 1). Of these, 3626 (34.5%) managed AF with a rhythm-control strategy prior to the visit, 5642 (53.7%) with a rate-control strategy, 1223 (11.7%) with no determined strategy, and 6 (<0.1%) with both strategies. The 6 patients using both strategies were not included in the analyses. The distribution of management strategy for each participating country in the study is shown in Table in S1 Table. Patients using a rhythm-control strategy were younger, with 21.4% aged > = 75 years (vs. 32.5% and 27.3% in those using a rate- control strategy or no strategy, respectively). For those patients using a rhythm-control strategy, 48.9%, 33.6%, and 12.1% of patients had paroxysmal, persistent, or permanent AF, respectively. In comparison, for those patients using a rate-control strategy, 8.7%, 15.4%, and 72.6% of patients had paroxysmal, persistent, or permanent AF, respectively. Corresponding numbers for patients with no strategy were in-between those of patients using a rhythm-control strategy and those of patients using a rate-control strategy. The time since first diagnosis of AF was more likely to have occurred ≥12 months previously for patients using a rate-control strategy (74.0%) than for those using a rhythm-control strategy (56.1%) or no strategy (32.4%).

**Table 1:** Patient characteristics according to AF management strategy prior to the visit.

	Rhythm-control Strategy (n = 3626)	Rate-control Strategy (n = 5642)	No strategy(n = 1223)	p-value^a^
Age in years, mean (SD)	64.8 (11.9)	68.0 (11.9)	65.7 (13.5)	<0.001
> = 75 years, %	21.4	32.5	27.3	<0.001
Male, %	56.4	55.8	58.9	0.52
Type of AF, %				<0.001
Paroxysmal	48.9	8.7	28.4	
Persistent	33.6	15.4	20.7	
Permanent	12.1	72.6	26.8	
First episode	5.3	3.3	24.1	
Time since first AF diagnosis				<0.001
<3 months	21.5	12.6	55.0	
3–6 months	8.9	5.0	4.7	
6–12 months	13.6	8.4	7.9	
> = 12 months	56.1	74.0	32.4	
**Cardiovascular risk factors and comorbidities**				
Hypertension	74.5	72.0	66.0	0.007
CHADS_2_ score > = 2	52.3	66.2	51.0	<0.001
Obesity (BMI > = 30 kg/m^2^)	34.0	32.6	29.5	0.16
Heart failure				<0.001
No heart failure or NYHA I	69.0	52.0	70.3	
NYHA II	21.6	28.8	17.4	
NYHA III or IV	9.4	19.2	12.3	
Left ventricular ejection fraction				<0.001
<35%	4.4	8.2	5.8	
35–50	14.4	22.4	17.4	
> = 50%	81.3	69.4	76.8	
Coronary artery disease	29.1	35.2	28.5	<0.001
Cerebrovascular disease	10.8	16.8	11.3	<0.001
Peripheral arterial disease	3.5	5.6	3.1	<0.001
Valvular heart disease	17.8	33.7	20.8	<0.001

^a^Rhythm vs. rate-control strategy.

AF, atrial fibrillation; BMI, body mass index; CHADS_2_, Cardiac failure, hypertension, age > = 75 years, diabetes, prior stroke [doubled]; NYHA, New York Heart Association; SD, standard deviation.

### Cardiovascular risk factors and comorbidities according to AF management strategy prior to the visit

In patients using a rate-control or a rhythm-control strategy prior to the visit, the proportion with hypertension (74.5% and 72.0%, respectively) and with obesity (34.0% and 32.6%, respectively) was similar. However, more patients using a rate-control than a rhythm-control strategy had coronary artery disease (35.2% vs. 29.1%, respectively), cerebrovascular disease (16.8% vs. 10.8%, respectively) or valvular heart disease (33.7% vs. 17.8%, respectively) (Table 1). Patients with more severe heart failure (NYHA III or IV) were more likely to be using a rate-control than a rhythm-control strategy (19.2% vs. 9.4%, respectively), while those with no heart failure/NYHA I were more likely to be using a rhythm-control than a rate-control strategy (69.0% vs. 52.0%, respectively) (Table 1).

### AF control and symptoms according to AF management strategy prior to visit

**Fig 1:**
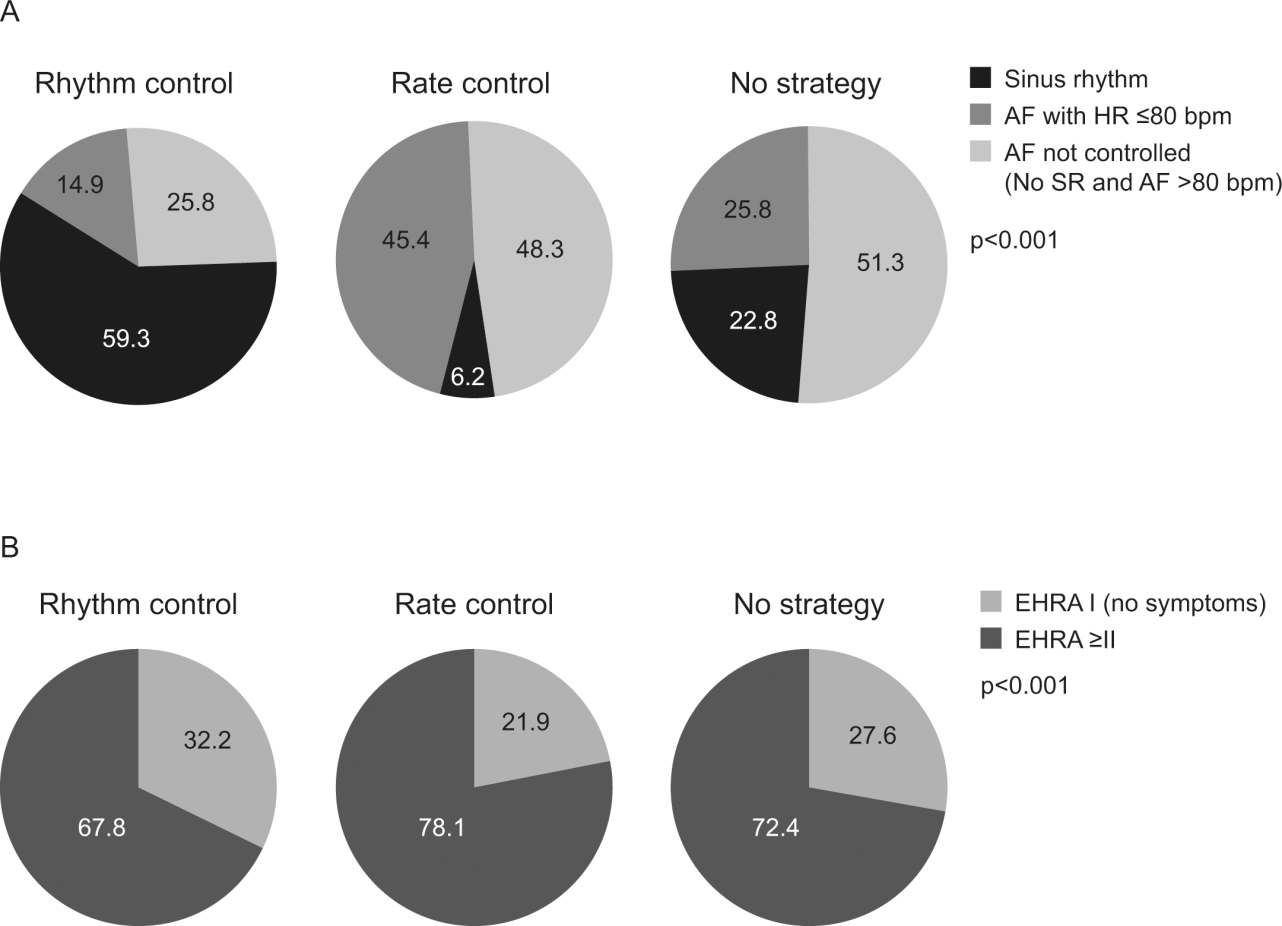
(A) AF control (at visit) and (B) AF symptoms (EHRA) (at visit) according to AF management strategy prior to the visit. p<0.001 (AF control); p<0.001 (AF symptoms). AF, atrial fibrillation; bpm, beats per minute; EHRA, European Heart Rhythm Association.

Fig 1A shows control of AF (assessed on the day of the visit) according to the AF management strategy used prior to the visit. Control of AF was evaluable in 91.8% (n = 9634/10,491) of the total population. In patients using a rhythm-control strategy (34.7%; n = 3340/9634), 74.2% were in sinus rhythm or in AF with a heart rate ≤80 bpm on a resting ECG. In comparison, in patients managed with a rate-control strategy (53.7%; n = 5178/9634), only 51.6% were in sinus rhythm or in AF with a heart rate < = 80 bpm on a resting ECG. A total of 11.6% (n = 1116/9634) were not using any clearly identified strategy, but 48.6% (n = 543/1116) of these had AF control.

Fig 1B shows EHRA symptom classification during the visit according to prior AF management strategy; these data were evaluable in 99.7% (n = 10,463/10,491) of patients. The proportion with AF-related symptoms (EHRA Class > = II) was 67.8% (n = 2452/3617) in patients using a rhythm-control strategy prior to the visit vs. 78.1% (n = 4396/5630) in patients using a rate-control strategy prior to the visit.

### Predictive factors for the choice of AF management strategy at end of visit

**Fig 2:**
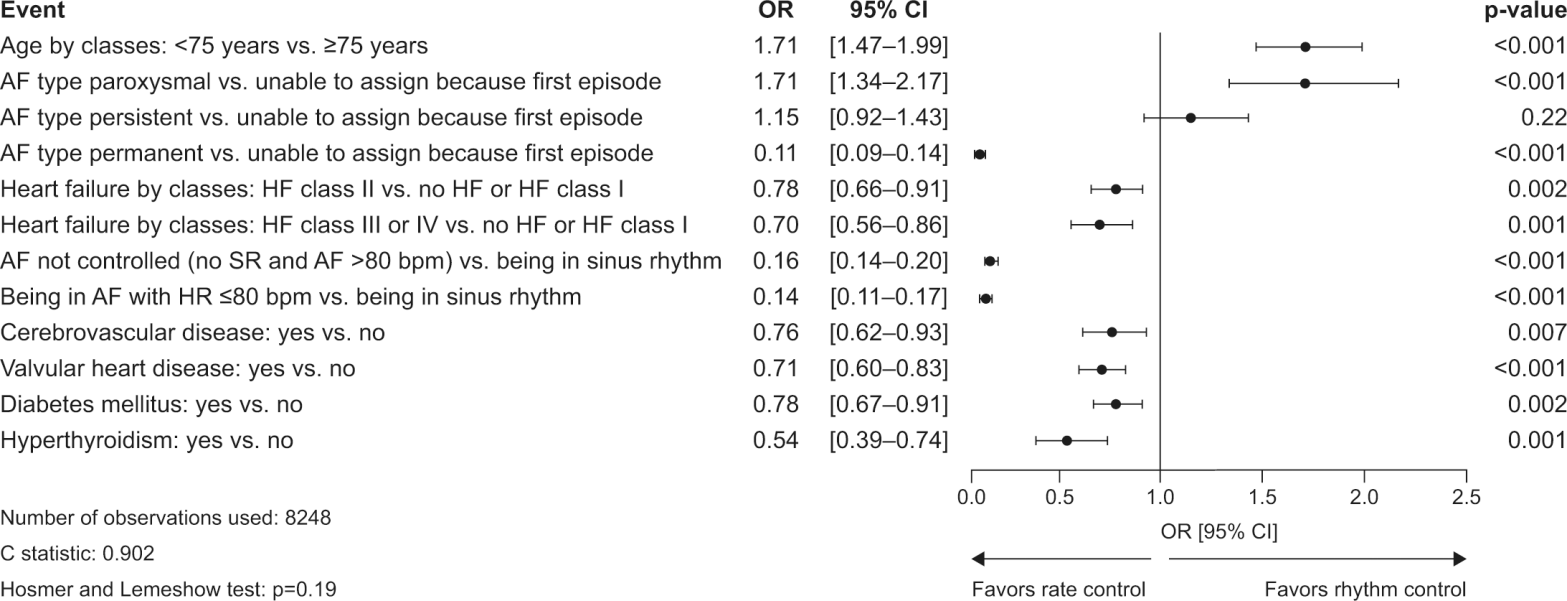
Multivariate logistic regression analysis: independent predictors for choice of management strategy at the end of the visit (rhythm-control strategy vs. rate-control strategy). AF, atrial fibrillation; BMI, body mass index; bpm, beats per minute; CI, confidence interval; EHRA, European Heart Rhythm Association; HF, heart failure; HR, heart rhythm; OR, odds ratio.

Based on the findings of the multivariate logistic regression analysis, after adjustment by country, independent predictors for choosing a rhythm-control strategy were paroxysmal AF, persistent AF, or age <75 years. Independent predictors for choosing a rate-control strategy were permanent AF; uncontrolled AF (vs. being in sinus rhythm); AF with heart rate < = 80 bpm (vs. being in sinus rhythm); or having cerebrovascular disease, diabetes, hyperthyroidism, valvular heart disease, or symptomatic heart failure (Fig 2).

### Change in AF management strategy at end of visit

Changes in AF management strategy were relatively infrequent in the AF patients surveyed. Among patients with controlled and uncontrolled AF, 14.0% and 21.8% of patients, respectively, had a change in management strategy at the end of the visit (15.1% among patients in sinus rhythm and 13.1% among patients in AF with a heart rate < = 80 bpm) (Table 2). A change in AF management strategy was more frequent for symptomatic patients (EHRA Class > = II) than for those without any symptoms (EHRA Class I) (17.5% vs. 14.8%, respectively). Similarly, for patients with uncontrolled AF, a change in management strategy was more frequent for symptomatic patients (EHRA Class > = II) than for those without any symptoms (EHRA Class I) (22.1% vs. 20.3%, respectively), and the change from no specific strategy to an AF management strategy was more frequent in symptomatic patients (EHRA Class > = II; 58.3%) (Table 2). Among the 468 patients with no determined strategy, uncontrolled AF and symptoms (EHRA Class > = 2), 11.3% remained in this undetermined strategy at the end of the visit.

**Table 2:** Change (%) in AF management strategy at the end of the visit.

AF control and symptoms (on day of visit)	Change (yes)	“No strategy” to “strategy”	Rhythm- to rate-control strategy	Rate- to rhythm-control strategy	Other change
AF controlled (n = 5704)	14.0	36.8	22.9	23.7	16.5
AF not controlled (n = 3961)	21.8	57.2	23.1	16.4	3.4
Sinus rhythm (n = 2559)	15.1	38.6	15.8	24.4	21.2
In AF with HR ≤80 bpm (n = 3145)	13.1	35.2	29.6	23.1	12.1
EHRA I (n = 2740)	14.8	42.6	23.6	21.7	12.1
EHRA ≥II (n = 7751)	17.5	48.9	22.8	19.6	8.7
AF not controlled, EHRA I (n = 706)	20.3	52.4	26.6	18.2	2.8
AF not controlled, EHRA > = II (n = 3245)	22.1	58.3	22.2	16.0	3.5

AF, atrial fibrillation; bpm, beats per minute; EHRA, European Heart Rhythm Association; HR, heart rate.

### Patient characteristics according to the AF management strategy at end of visit

At the end of the visit, 3909 (37.2%), 6036 (57.5%), and 533 (5.1%) patients were using a rhythm-control, rate-control, or no strategy, respectively (Table 3). Patients using a rhythm-control strategy at the end of the visit were younger, with only 20.4% aged > = 75 years. A greater proportion of these patients had paroxysmal AF (49.3%) than either persistent (33.4%) or permanent (9.0%) AF. In comparison, a greater proportion of patients using a rate-control strategy had permanent AF (71.2%) than either paroxysmal (8.5%) or persistent (15.3%) AF.

**Table 3:** Patient characteristics according to AF management strategy at the end of the visit.

	Rhythm-control Strategy (n = 3909)	Rate-control Strategy (n = 6036)	p-value^a^
Age in years, mean (SD)	64.5 (11.9)	68.0 (12.0)	<0.001
> = 75 years, %	20.4	32.5	<0.001
Male, %	57.3	55.1	0.03
Type of AF, %			<0.001
Paroxysmal	49.3	8.5	
Persistent	33.4	15.3	
Permanent	9.0	71.2	
Unable to assign because first episode	8.2	5.0	
Time since first AF diagnosis			<0.001
<3 months	28.2	15.3	
3–6 months	8.5	4.8	
6–12 months	12.7	8.3	
>12 months	50.6	71.7	
Hypertension	72.9	72.2	0.48
CHADS_2_ score > = 2	50.5	66.2	<0.001
Obesity (BMI > = 30 kg/m^2^)	33.0	32.9	0.85
Diabetes mellitus	18.2	23.7	<0.001
Heart failure			<0.001
No heart failure or NYHA I	70.0	52.2	
NYHA II	20.8	28.5	
NYHA III or IV	9.2	19.2	
Left ventricular ejection fraction			<0.001
<35%	4.3	8.3	
35–50	14.8	22.0	
> = 50%	80.8	69.7	
Coronary artery disease	29.0	34.9	<0.001
Cerebrovascular disease	10.1	16.6	<0.001
Peripheral arterial disease	3.1	5.6	<0.001
Valvular heart disease	16.5	33.3	<0.001

^a^Rhythm-control vs. rate-control strategy.

AF, atrial fibrillation; BMI, body mass index; CHADS_2_, Cardiac failure, hypertension, age ≥75 years, diabetes, prior stroke [doubled]; NYHA, New York Heart Association; SD, standard deviation.

### Cardiovascular risk factors and comorbidities according to AF management strategy at end of visit

The pattern of cardiovascular risk factors and comorbidities according to AF management strategy at the end of the visit was consistent with that prior to the visit. For example, the proportion of patients with hypertension or obesity remained similar both within and between groups, and more patients using a rate-control strategy had coronary artery disease, cerebrovascular disease, or valvular heart disease (Table 3).

### AF management strategy at end of visit according to AF control and symptoms

**Fig 3:**
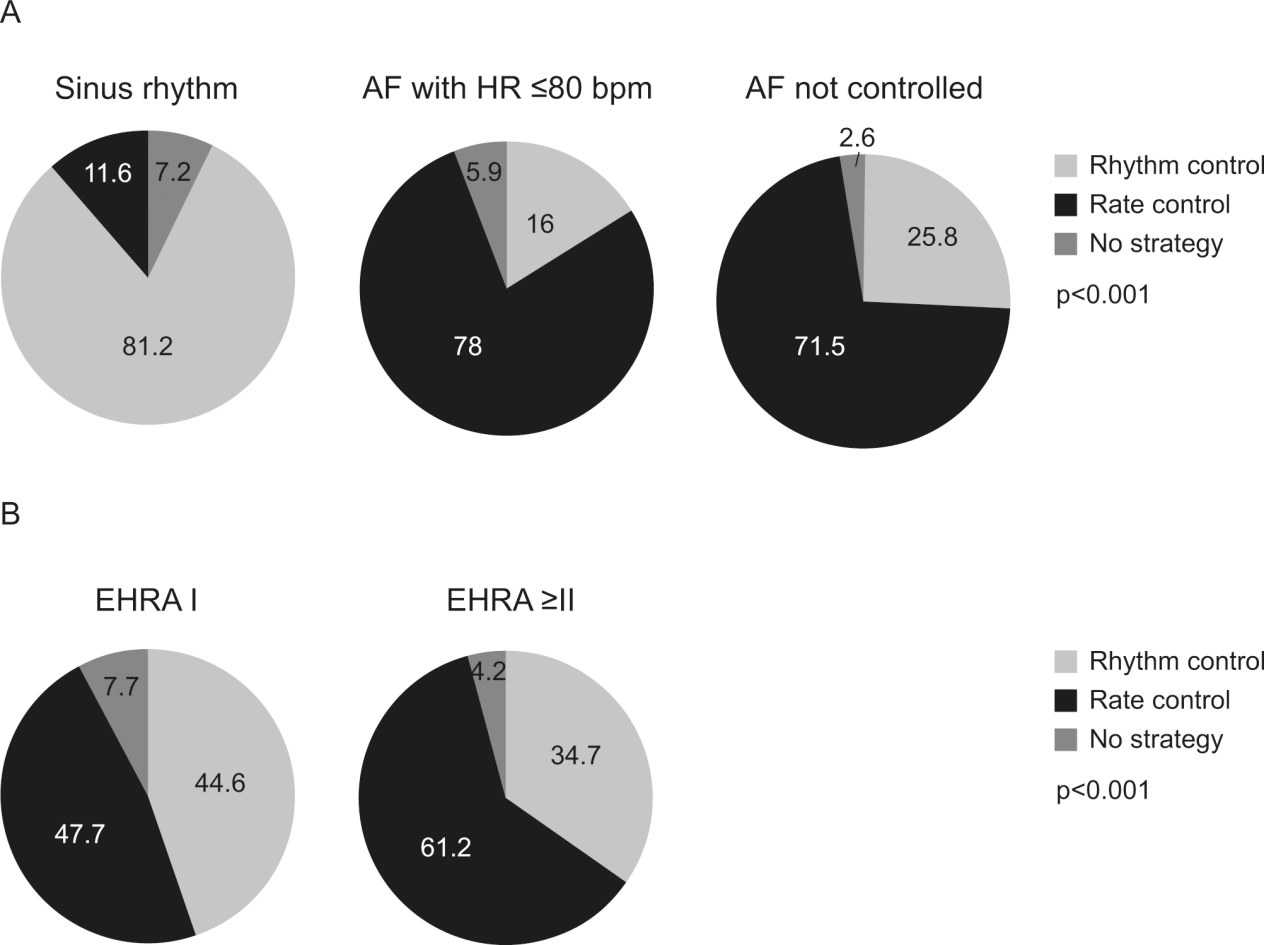
AF management strategy at end of visit according to (A) AF control (at visit) and (B) AF symptoms (EHRA) (at visit). p<0.001 (AF control); p<0.001 (AF symptoms). AF, atrial fibrillation; bpm, beats per minute; EHRA, European Heart Rhythm Association.

A total of 9621 patients assessed for AF control had data related to AF management at the end of the visit. For patients who were in sinus rhythm as assessed during the visit, the majority (81.2%; n = 2074/2553) were using a rhythm-control strategy at the end of the visit. Conversely, for patients who were in AF with a heart rate ≤80 bpm as assessed during the visit, the majority (78.0%; n = 2446/3135) were using a rate-control strategy at the end of the visit. Similarly, for patients with uncontrolled AF as assessed during the visit, 71.5% (n = 2814/3933) were using a rate-control strategy at the end of the visit (Fig 3A).

In patients with AF-related symptoms (EHRA Class > = II) as assessed during the visit, (Fig 3B), the majority were using a rate-control strategy (61.2%; n = 4719/7716) rather than a rhythm-control strategy (34.7%; n = 2676/7716) or no strategy (4.2%; n = 321/7716) at the end of the visit.

## Discussion

The major finding of this international cross-sectional survey is that a rate-control strategy appears to be used more commonly than a rhythm-control strategy for AF in a routine clinical practice setting. Another important finding is that physicians did not clearly select either strategy for AF management in 11.7% of patients. Patients using a rate-control strategy were generally older; more symptomatic; and more likely to have coronary artery, cerebrovascular, or valvular heart disease than those who used a rhythm-control strategy.

Interestingly, far more patients in the present survey used a rate-control strategy than in the previous Euro Heart Survey (54% vs. 27%, respectively) [10]. This is due to several factors: Compared with the Euro Heart Survey, the present survey had a larger proportion of patients with permanent AF, included many countries outside of Europe, and randomly selected the participating physicians, thereby providing a more generalizable picture of AF management.

To date, clinical trials and meta-analyses have been unable to demonstrate outcome benefits from a rhythm-control strategy [2,11,12]. For example, in the Atrial Fibrillation Follow-up Investigation of Rhythm Management (AFFIRM) trial, the percentage of patients requiring hospitalization was significantly lower in patients using a rate-control strategy than those using a rhythm-control strategy (p<0.001) as was the incidence of torsade de pointes (p = 0.007) [2]. For patients with heart failure in particular, the pre-specified subgroup analysis of the AFFIRM study did not support the use of rhythm-control strategy [2]. In the AF-CHF trial enrolling patients with AF and heart failure, a routine strategy of rhythm control did not reduce the rate of death from CV cause and worsening of heart failure, as compared with a rate-control strategy [11]. In terms of stroke prevention in the AFFIRM study, the rhythm-control strategy resulted in numerically more patients suffering from ischemic stroke [2]. In a meta-analysis of rhythm control vs rate control strategy, the proportion of patients experiencing an ischemic stroke was similar between the rate-control and rhythm-control groups [13].

There is some evidence in favor of a rhythm-control strategy in some observational studies [14,15,16], and in post-hoc analyses of the AFFIRM trial, in which patients who were maintained in sinus rhythm had better survival rates [17]. In addition, in the REgistry on Cardiac rhythm disORDers assessing the control of Atrial Fibrillation (RECORD-AF) registry [18], the use of an early rhythm-control strategy was associated with a lower risk of AF progression.

European and US practice guidelines recommend an initial rate-control strategy for patients with minimal or no symptoms [19,20]. In the present survey, among asymptomatic patients (EHRA I), the majority (47.7%) were managed with a rate-control strategy; however, a similar proportion (44.6%) were managed with a rhythm-control strategy. This suggests that many patients with AF are not being treated in a fashion consistent with the guidelines.

Our multivariate logistic regression analysis that elicited the independent predictors for choice of management strategy confirmed prior observations [21,22] that a rhythm-control strategy was generally used in patients who were younger (<75 years) or had paroxysmal or persistent AF; whereas the presence of structural heart disease, or comorbidities such as diabetes or hyperthyroidism, were better correlated with use of a rate-control strategy.

In this cross-sectional survey, changes in management strategy were infrequent although, as would be expected, they were slightly more common in symptomatic patients and in those with uncontrolled AF. The majority of patients with symptomatic (82.5%), uncontrolled (78.2%) or symptomatic uncontrolled (77.9%) AF did not undergo a change in management strategy. Among the 468 patients with no determined strategy, uncontrolled AF and symptoms (EHRA Class > = 2), 11.3% remained in this undetermined strategy at the end of the visit. These findings may reflect medical inertia, as well as lingering uncertainties regarding the optimal management strategy for each patient.

### Limitations

The present report should be interpreted cautiously, given its observational and cross-sectional nature. Patients were not randomly assigned to different strategies. Despite the wide geographic scope of this study, it does not include Central Africa or the United States and Canada, where there might be major differences in patient characteristics and preferred management strategies. Additionally, there is a sizeable group of patients for whom no clear strategy was chosen by the treating physician, reflecting the need for further clarification and education on AF management.

Frequency of attack of AF may impact on the strategy which was undertaken. But in the daily practice, this might not necessarily be true, and other clinical characteristics, including symptoms, should be considered. The inclusion criteria in this study was the same as that in the EURO Heart Survey [10] and the RECORD-AF registry [8]. Neither of these 2 studies could provide information regarding the frequency of AF and management strategy. Instead of studying the impact of frequency of episodes on management strategy, the main purpose of this study was to show the correlates of strategy with patients' clinical characteristics, AF control, and symptom burden in routine clinical practice. Future studies which contain more comprehensive description of AF burden before entering may be needed to answer this question.

The choice of a specific resting heart rate to define adequate rate control is somewhat arbitrary. Previous AF guidelines defined adequate resting heart rate control as 60 to 80 bpm [23], and a rate of <80 bpm was used by the AFFIRM study investigators [2]. Thus, 80 bpm was chosen as the protocol definition of “controlled” AF in the present survey [1].

In conclusion, in the RealiseAF survey reflecting routine clinical practice, a rate-control strategy was most frequently used, especially in patients with cardiovascular comorbidities. A change in the management strategy of AF patients appears to be uncommon in clinical practice, suggesting that awareness among physicians regarding optimal management strategies for AF could be improved.

## Supporting Information

S1 Table Distribution of AF management strategy prior to the visit for each country. (DOCX)
