# Supplementary material for: Atrial Fibrillation Management Strategies in Routine Clinical Practice: Insights from the International RealiseAF Survey
Source: PLoS One. 2016 Jan 22;11(1):e0147536. doi: 10.1371/journal.pone.0147536 (PMC4723091; doi:10.1371/journal.pone.0147536)
Supplement: S1 Table — (DOCX) [file pone.0147536.s001.docx]

**S1 Table**

Distribution of AF management strategy prior to the visit for each country.

|  | **Therapeutic strategy chosen for AF prior to the visit** | | |
| --- | --- | --- | --- |
| **Country** | **Rhythm control,**  **n/N (%)** | **Rate control,**  **n/N (%)** | **No strategy, n/N (%)** |
| **All** | **3626/10,491 (34.6)** | **5642/10,491 (53.8)** | **1223/10,491 (11.7)** |
| Algeria | 63/310 (20.3) | 198/310(63.9) | 49/310 (15.8) |
| Azerbaijan | 51/150 (34.0) | 47/150 (31.3) | 52/150 (34.7) |
| Belgium | 131/306 (42.8) | 147/306 (48.0) | 28/306 (9.2) |
| Bulgaria | 134/450 (29.8) | 283/450 (62.9) | 33/450 (7.3) |
| Czech Republic | 108/280 (38.6) | 156/280 (55.7) | 16/280 (5.7) |
| Egypt | 103/458 (22.5) | 294/458 (64.2) | 61/458 (13.3) |
| Germany | 476/1063 (44.8) | 470/1063 (44.2) | 117/1063 (11.0) |
| Hungary | 144/506 (28.5) | 313/506 (61.9) | 49/506 (9.7) |
| India | 103/292 (35.3) | 135/292 (46.2) | 54/292 (18.5) |
| Ireland | 72/224 (32.1) | 128/224 (57.1) | 24/224 (10.7) |
| Italy | 118/253 (46.6) | 109/253 (43.1) | 26/253 (10.3) |
| Lebanon | 38/190 (20.0) | 108/190 (56.8) | 44/190 (23.2) |
| Lithuania | 230/452 (50.9) | 179/452 (39.6) | 43/452 (9.5) |
| Mexico | 32/168 (19.0) | 126/168 (75.0) | 10/168 (6.0) |
| Morocco | 79/250 (31.6) | 103/250 (41.2) | 68/250 (27.2) |
| Portugal | 68/165 (41.2) | 87/165 (52.7) | 10/165 (6.1) |
| Russia | 313/750 (41.7) | 389/750 (51.9) | 48/750 (6.4) |
| Slovakia | 174/437 (39.8) | 228/437 (52.2) | 35/437 (8.0) |
| Spain | 152/486 (31.3) | 296/486 (60.9) | 38/486 (7.8) |
| Sweden | 135/264 (51.1) | 112/264 (42.4) | 17/264 (6.4) |
| Switzerland | 121/401 (30.2) | 203/401 (50.6) | 77/401 (19.2) |
| Taiwan | 162/742 (21.8) | 486/742 (65.5) | 94/742 (12.7) |
| Tunisia | 166/471 (35.2) | 227/471 (48.2) | 78/471 (16.6) |
| Turkey | 78/510 (15.3) | 355/510 (69.6) | 77/510 (15.1) |
| Ukraine | 281/700 (40.1) | 358/700 (51.1) | 61/700 (8.7) |
| Venezuela | 94/213 (44.1) | 105/213 (49.3) | 14/213 (6.6) |
